# Assessment of Xenoestrogens Using Three Distinct Estrogen Receptors and the Zebrafish Brain Aromatase Gene in a Highly Responsive Glial Cell System

**DOI:** 10.1289/ehp.8141

**Published:** 2005-12-08

**Authors:** Yann Le Page, Martin Scholze, Olivier Kah, Farzad Pakdel

**Affiliations:** 1 Endocrinologie Moléculaire de la Reproduction, Université de Rennes, Rennes, France; 2 Centre for Toxicology, School of Pharmacy, University of London, London, United Kingdom

**Keywords:** aromatase gene, brain, endocrine disruptors, estrogen receptors, glial cells, xenoestrogens, zebrafish

## Abstract

The brain cytochrome P450 aromatase (Aro-B) in zebrafish is expressed in radial glial cells and is strongly stimulated by estrogens (E_2_); thus, it can be used *in vivo* as a biomarker of xenoestrogen effects on the central nervous system. By quantitative real-time polymerase chain reaction, we first confirmed that the expression of *Aro-B* gene is robustly stimulated in juvenile zebrafish exposed to several xenoestrogens. To investigate the impact of environmental estrogenic chemicals on distinct estrogen receptor (ER) activity, we developed a glial cell-based assay using *Aro-B* as the target gene. To this end, the ER-negative glial cell line U251-MG was transfected with the three zebrafish ER subtypes and the Aro-B promoter linked to a luciferase reporter gene. E_2_ treatment of U251-MG glial cells cotransfected with zebrafish ER-α and the Aro-B promoter–luciferase reporter resulted in a 60- to 80-fold stimulation of luciferase activity. The detection limit was < 0.05 nM, and the EC_50_ (median effective concentration) was 1.4 nM. Interestingly, in this glial cell context, maximal induction achieved with the Aro-B reporter was three times greater than that observed with a classical estrogen-response-element reporter gene (*ERE-tk-Luc*). Dose–response analyses with ethynylestradiol (EE_2_), estrone (E_1_), α-zeralenol, and genistein showed that estrogenic potency of these agents markedly differed depending on the ER subtype in the assay. Moreover, the combination of these agents showed an additive effect according to the concept of concentration addition. This confirmed that the combined additive effect of the xenoestrogens leads to an enhancement of the estrogenic potency, even when each single agent might be present at low effect concentrations. In conclusion, we demonstrate that our bioassay provides a fast, reliable, sensitive, and efficient test for evaluating estrogenic potency of endocrine disruptors on ER subtypes in a glial context.

In all vertebrate species, endogenous estrogens (E_2_) play a crucial role in the development, maintenance, and function of female and male reproductive tracts. In addition, the importance of E_2_ in many other tissues such as bone, the cardiovascular system, and the central nervous system is well documented (Emmen and Korach 2003; Enmark et al. 1997; Maggi et al. 2000; McDonnell 2003). In mammals, two estrogen receptors (ER-α and ER-β) generated from two distinct genes have been characterized (Green et al. 1986; Kuiper et al. 1996). These receptors show partially distinct expression patterns, and their activities are modulated differently by some ligands called selective ER modulators (SERMs) (Gustafsson 1998; Katzenellenbogen et al. 2000). Among the compounds affecting ER signaling are an increasing number of man-made substances or natural phytoestrogens with estrogenic or antiestrogenic properties.

Indeed, in the 1990s, the appearance of adverse reproductive effects in aquatic and wildlife species living within or near contaminated areas was reported in scientific literature (Colborn et al. 1993; Giesy et al. 1994; Guillette et al. 1994; Sumpter and Jobling 1993). To determine whether environmental contaminants could alter the function of the endocrine systems, male wild fish in U.K. rivers were exposed to effluents from waste-water treatment works (Sumpter 1995; Sumpter and Jobling 1995). Male fish in these studies showed intersex phenomena (female ovarian tissue within the testes) and produced vitellogenin, a protein required for egg yolk production in females. Moreover, in a study by Sharpe et al. (1995), the exposure of rats to xenoestrogens during gestation and lactation resulted in reduced testicular size and sperm production. In parallel, several *in vitro* and cell-based assays showed that some substances generated from pesticides, herbicides, plastic components, heavy metals, pharmaceuticals, and so forth, have estrogenic or antiestrogenic activity (Balaguer et al. 1996; Flouriot et al. 1995; Petit et al. 1997; Soto et al. 1991, 1995). Together, these observations led to the conclusion that environmental contaminants may interfere with normal hormonal processes and act as estrogenic or antiestrogenic chemicals (Colborn et al. 1993; Sharpe and Skakkebaek 1993; Sohoni and Sumpter 1998; Sonnenschein and Soto 1998; Sumpter 1995).

Various fish species, particularly zebrafish, are commonly used as model organisms to analyze the impact of endocrine disruptors (EDs) found in the environment. In fish, the existence of three rather than two ERs (Hawkins et al. 2000; Menuet et al. 2002), characterized as ER-α, ER-β1, and ER-β2, indicates that the mechanism of action of estrogens and environmental estrogenic chemicals may be more complex than previously envisioned. We reported previously that zebrafish ERs (zfERs) are predominantly expressed in the reproductive tissues and also in the brain, where the three ERs showed partially overlapping patterns (Menuet et al. 2002). The brain of teleost fish is characterized by an important aromatase activity that is due to the expression of a brain-specific aromatase gene, encoding cytochrome P450 aromatase B (*Aro-B*) (Tchoudakova et al. 2001). Interestingly, expression of *Aro-B* is restricted to radial glial cells (Forlano et al. 2001; Menuet et al. 2003, 2005), and its expression is up-regulated by E_2_ (Kazeto et al. 2004; Kishida and Callard 2001). We have recently shown *in vivo* and *in vitro* that, in zebrafish, this E_2_ up-regulation of Aro-B expression requires the presence of functional ERs and occurs only in glial cell contexts (Menuet et al. 2005). Aro-B is a crucial enzyme that aromatizes androgens into estrogens, and this local production of E_2_ is likely to be very important for the development, growth, and sex differentiation of the brain. There is also an indication that the *Aro-B* gene can be used as a sensitive marker of the effects of xenoestrogens on the central nervous system during embryogenesis (Kishida et al. 2001) and in zebrafish juveniles (Kazeto et al. 2004). However, to date, there is no report on the potential transcriptional effects of xenoestrogens on the Aro-B promoter due to the lack of appropriate cell-based assays.

Recently, we linked 500 bp of the proximal promoter region of zebrafish *Aro-B* gene to the luciferase reporter gene. Transfection experiments with the promoter-luciferase reporter in different cell contexts showed that, similar to the *in vivo* situation, full E_2_ up-regulation of the *Aro-B* gene is restricted to glial cell lines, such as the human glial cell line U251-MG (Menuet et al. 2005).

In this study, we tested the impact of several xenoestrogens, individually or in mixture, on the transcriptional activity of three distinct ERs in this glial cell context. To achieve this, we used the ER-negative glial cell line U251-MG to express each ER subtype and the endogenous zebrafish Aro-B promoter as the reporter gene. We tested low-dose and mixtures of EE_2_, E_1_, α-zeralenol, and genistein together with E_2_ (positive control) and ethanol (solvent, negative control). We chose these chemicals because they previously have been characterized as potent and environmentally relevant xenoestrogens (Kazeto et al. 2004; Le Guevel and Pakdel 2001; Thorpe et al. 2003). Indeed, about 80% of estrogenic activity in the U.K. domestic effluent corresponded to natural and synthetic estrogens, such as E_2_, E_1_, and EE_2_ (Rodgers-Gray et al. 2001; Thorpe et al. 2003). Our results show that all these chemicals stimulate *Aro-B* gene expression *in vivo*. Moreover, in the glial cell system, all three zfERs strongly activate the Aro-B promoter. ER-α was 2- to 3-fold more efficient than ER-β2 and 3- to 5-fold more efficient than ER-β1. Although the xenoestrogens tested did not change ER efficiency in activating the *Aro-B* reporter gene, we found that EE_2_ and genistein are more sensitive to ER-β subtypes than to ER-α. Dose–response curves with the mixture of five estrogenic chemicals showed that combination of these agents results in a concentration-additive effect in our reconstituted glial model.

## Materials and Methods

### In vitro *transcription/translation of zfERs.*

To synthesize zfER proteins, we performed an *in vitro* translation reaction using 1 μg of each ER expression vector and T7 RNA polymerase in a rabbit reticulocyte lysate. The reaction was performed in the presence of ^35^S-methionine at 30°C for 90 min as recommended by the supplier (Quick TNT; Promega, Madison, WI, USA).

### Cell culture and transfection.

U251-MG cells were maintained at 37°C in 5% CO_2_ atmosphere in phenol red–free Dulbecco's Modified Eagle’s Medium (DMEM-F12; Sigma-Aldrich, St. Louis, MO, USA) supplemented with 8% fetal calf serum (FCS; Life Technologies, Carlsbad, CA, USA). The medium is also supplemented with 2 mM l-glutamine (Gibco, Carlsbad, CA, USA), 20 U/mL penicillin, 20 μg/mL streptomycin, and 50 ng/mL amphotericin B (Gibco). For transfection experiments, cells were plated in 24-well plates at a density of 0.2 × 10^5^ cells/mL. In each well, 25 ng of expression vector, 25 ng of cytomegalovirus–β-galactosidase control plasmid and 150 ng of luciferase reporter construct were transfected with FuGENE 6 transfection reagent (Roche, Basel, Switzerland). After one night, medium was replaced with fresh DMEM-F12 containing 2% charcoal/dextran FCS with xenoestrogen or vehicle. The luciferase activities were assayed 48 hr later using the luciferase assay system (Promega). We used β-galactosidase activity to normalize transfection efficiency in all experiments. Each experiment was performed at least in triplicate.

### Plasmid construction and site-direct mutagenesis.

The zfER-α, zfER-β1, and zfER-β2 expression vectors correspond to Topo-pcDNA3 expression vector (Invitrogen, San Diego, CA, USA), containing the coding regions of each receptor cDNA as previously described (Menuet et al. 2002). The estrogen response element (ERE)–thymidine kinase–luciferase reporter gene (*ERE-tk-Luc*) consists of a consensus ERE with a minimal thymidine kinase promoter driving firefly luciferase activity. This well-characterized ERE reporter responds to all ER subtypes in several cell lines (Ackermann et al. 2002; Menuet et al. 2002, 2005; Metivier et al. 2001).

The *Aro-B* reporter gene consists of 500 bp of the proximal promoter region of zebrafish cytochrome P450 *19b* gene, containing an ERE, coupled to the luciferase reporter gene. This reporter gene was described previously by Menuet et al. (2005).

The *Aro-B* mutated reporter construct (*Aro-B mut*) is similar to the Aro-B reporter wild type except that the ERE was mutated by site-directed mutagenesis. We used the QuickChange site-directed mutagenesis kit from Stratagene (La Jolla, CA, USA) and the following primers: 5′-GGTTCTGAATCAGTCTGAAATGCCTTCATTAAAAGC-3′ and 5′-AATGAAGGCATTTCAGACTGATTCAGAACCAAACC-3′. Each construct was sequenced by the PRISM (Perkin Elmer Applied Biosystems, Foster City, CA, USA) ready reaction big dye terminator cycle sequencing protocol.

### Zebrafish exposure to xenoestrogens and RNA extraction.

All zebrafish were from our local facility. They are raised in 28.5°C recirculated water and kept under a 12-hr dark/12-hr light cycle. Animals were treated in agreement with the European Union regulations concerning the protection of experimental animals. At least 10 juvenile zebrafish 18–21 days of age were exposed to xenoestrogens or vehicle for 3 days in glass tanks containing 100 mL embryo medium (15 mM NaCl, 0.5 mM KCl, 1 mM MgSO_4_, 1 mM CaCl_2_, 0.15 mM KH_2_PO_4_, 0.05 mM Na_2_HPO_4_, 0.7 mM NaHCO_3_, 10^−5^% methylene blue; pH 7.5). The medium was maintained at 26°C and replaced every day. After exposure, 10 zebrafish were sonicated together (10 sec, three times) in 1 mL Trizol Reagent (Gibco), and total RNA was extracted according to the manufacturer’s protocol.

### Quantitative real-time PCR.

Reverse transcription was carried out by incubating 2 μg total RNA with 5 mM random hexamer oligonucleotides, 10 mM DTT, 2.5 mM dNTPs and 100 U MMLV-RT (Promega) in the appropriate buffer for 30 min at 37°C and 15 min at 42°C. Polymerase chain reaction (PCR) reactions were performed in an iCycler thermocycler coupled to the MyiQ detector (Bio-Rad. Hercules, CA, USA) using iQ SYBR-Green Supermix (Bio-Rad) according to the manufacturer’s protocol. The following primers were used: Aro-B reverse transcriptase (RT)-up 5′-TCGGCACGGCGTGCAACTAC-3′, Aro-B RT-down 5′-CATACCTATGCATTGCAGACC-3′, GAPDH-up 5′-GAGCACCAGGTTGTGTCCA-3′, GAPDH-down 5′-TGTCATACCATGTGACCAGCTT-3′. Expression levels of GAPDH mRNA were used to normalize the expression levels of Aro-B mRNA. Melting curve and PCR efficiency analyses were performed to confirm a correct amplification. Each experiment was performed at least twice in triplicate.

### Concentration effect analyses.

We determined concentration–response relationships for the single compounds and for the mixtures using the best-fit approach described by Scholze et al. (2001). We used this information to calculate predicted mixture effects, with a ratio proportional to equieffective concentrations producing an effect of 30% for ER-α expression. The concept of concentration addition was used; for a detailed description, see Rajapakse et al. (2004). The statistical uncertainties for the predicted mean effect were expressed as 95% confidence belt and determined by using the bootstrap method (Efron and Tibshirani 1993).

## Results

### *Aro-B is a highly sensitive biomarker of xenoestrogens* in vivo.

**Figure 1:**
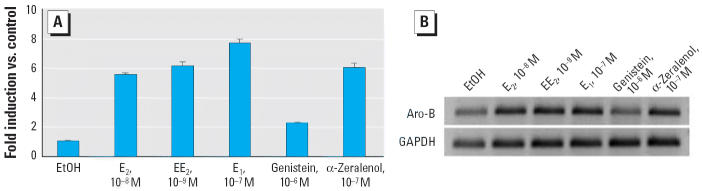
Environmental estrogenic chemicals stimulate the expression of Aro-B in zebrafish juveniles. At least 10 juvenile zebrafish 18–21 days of age were exposed to ethanol solvent (EtOH), 10 nM E_2_, 1 nM EE_2_, 100 nM E_1_, 100 nM α-zeralenol, or 1 μM genistein. (*A*) Expression of Aro-B measured in triplicate by real-time quantitative RT-PCR of total RNA prepared from pooled animals. Fold induction was expressed relative to the solvent; data are presented as mean ± SE of two separate exposures. (*B*) DNA fragment amplified by PCR for Aro-B and GAPDH (internal control) migrated at the expected sizes on the agarose gel, stained by ethidium bromide.

We tested the ability of individual chemicals to stimulate the expression of zebrafish brain Aro-B *in vivo*. Zebrafish juveniles, 18–21 days of age, were exposed for 3 days to E_2_ (10 nM), EE_2_ (1 nM), E_1_ (100 nM), α-zeralenol (100 nM), and genistein (1 μM), according to the relative estrogenicity of those chemicals. For each treatment, we used a pool of 10 juveniles, and we prepared total RNA from whole bodies. Figure 1 shows the expression of Aro-B measured by real-time quantitative RT-PCR experiments. As we expected, E_2_, EE_2_, E_1_, and α-zeralenol robustly stimulated the expression of Aro-B, whereas the expression of GAPDH—used as an internal control—remained unchanged. In these experiments, the fold stimulation of the *Aro-B* gene by xenoestrogens was about six to eight times that of the solvent control. Surprisingly, genistein is less potent than other chemicals, although we used a relatively high concentration (Figure 1).

### Estrogenic responsiveness of the reconstituted glial cell model.

**Figure 2:**
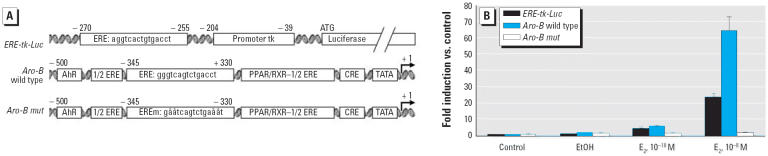
*Aro-B* reporter gene up-regulation by E_2_ in the glial cell line U251-MG. (*A*) Schematic representation of the three luciferase reporter constructs used (see “Materials and Methods” for description). (*B*) Fold induction in U251-MG cells transfected with empty expression vector (control) or zfER-α expression vector together with *ERE-tk-Luc*, *Aro-B*, or *Aro-B mut* constructs. Data are expressed as fold induction relative to control; each experiment was repeated at least twice in triplicate.

The ER-negative glial cell line U251-MG was transfected with zfER-α expression vector together with the *ERE-tk-Luc* reporter gene, the *Aro-B* reporter gene, or the *Aro-B mut* reporter gene. The sensitivity of the assay was tested with 0.1 and 10 nM E_2_ for 48 hr in 24-well plates (Figure 2). Relative to the cell controls (without ER expression vector), 10 nM E_2_ increased luciferase activity 22-fold from the *ERE-tk-Luc* reporter gene, whereas it increased luciferase activity 65-fold from the *Aro-B* reporter gene. As demonstrated by the *Aro-B mut* gene, the estrogenic effect of E_2_ required the integrity of the ERE sequence within the Aro-B promoter. Indeed, site-directed mutagenesis of this ERE completely abolished E_2_ stimulation of the *Aro-B* reporter gene.

**Figure 3:**
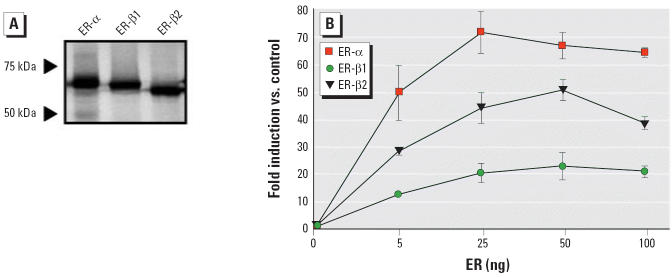
Examination of receptor concentration on E_2_ stimulation of *Aro-B* reporter gene. (*A*) zfERs produced as ^35^S-methionine–labeled proteins in a rabbit reticulocyte lysate and visualized by autoradiography after SDS-PAGE. (*B*) Dose effect of ERs in U251-MG cells transfected with the *Aro-B* reporter gene and increasing amounts of zfER expression vectors. Cells were treated with or without E_2_ (10^−8^ M) for 48 hr before luciferase activity was measured. Data are expressed as fold induction relative to empty vector (control).

Figure 3A shows that all three receptors were correctly expressed *in vitro* with a molecular mass of approximately 65 kDa in the rabbit reticulocyte lysate system.

To test whether receptor concentration could affect E_2_ stimulation of the *Aro-B* reporter gene differently in U251-MG cells, increasing amounts of zfER expression vectors were tested. Figure 3B shows that E_2_ stimulation of luciferase activity mediated by each ER corresponds to a distinct profile depending on zfER subtype. These profiles were not modified when receptor concentration was increased.

### Dose–response analysis of individual chemicals in the reconstituted glial cell model.

**Figure 4:**
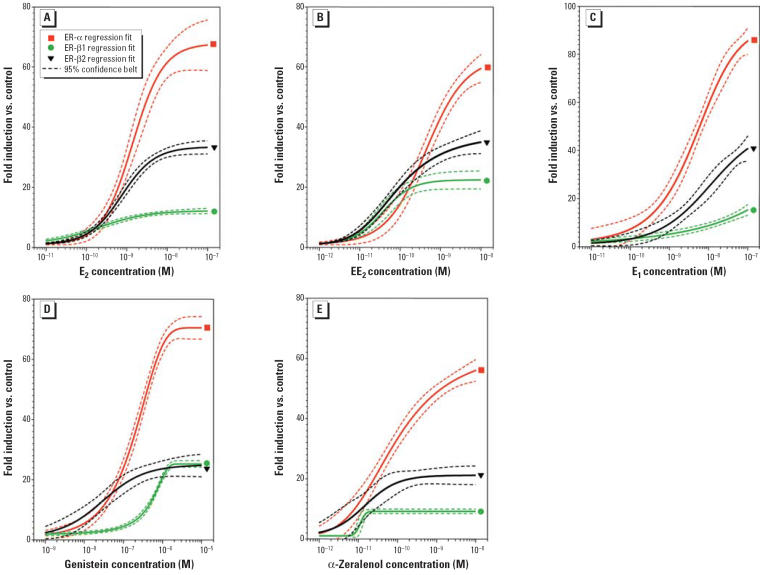
Dose-dependent effect of E_2_ and xenoestrogens on the transcriptional activation of zfERs in U251-MG cells transfected with the *Aro-B* reporter gene and the zfER expression vectors. Cells were treated with increasing concentrations of (*A*) E_2_ (10^−11^ M to 10^−7^ M), (*B*) EE_2_ (10^−12^ M to 10^−8^ M), (*C*) E_1_ (10^−11^ M to 10^−7^ M), (*D*) genistein (10^−9^ M to 10^−5^ M), or (*E*) α-zeralenol (10^−12^ M to 10^−8^ M). Data are expressed as fold induction relative to empty vector (control) from at least three experiments.

The glial cell line U251-MG was transfected with each zfER subtype (ER-α, ER-β1, and ER-β2) expression vector together with *Aro-B* wild-type reporter gene. Cells were treated with E_2_, EE_2_, E_1_, genistein, and α-zeralenol (Figure 4). We tested seven concentrations of each chemical, ranging from picomolar to micromolar. Estrogenic activity of each chemical was analyzed by the three ER subtypes and is represented as fold induction of luciferase activity versus control (luciferase reporter gene without ER). In all cases, ER-α stimulated 60-to 80-fold luciferase activity, whereas the maximum stimulation of the *Aro-B* reporter gene by ER-β1 and ER-β2 was two to six times lower (Figure 4, Table 1). ER-β2 stimulated 20- to 40-fold luciferase activity, whereas ER-β1 stimulated *Aro-B* reporter gene 10- to 20-fold. Table 1 shows the EC_50_ (median effective concentration) of different chemicals calculated for each ER subtype from the dose–response curves. Interestingly, the EC_50_ values of EE_2_ and genistein were lower for ER-β2 than those calculated for ER-α. In contrast, the EC_50_ of E_1_ was lower for ER-α than for ER-β2. Table 1 also shows the detection limit, arbitrarily fixed at 2-fold the basal activity and maximum induction for each chemical.

**Figure 5:**
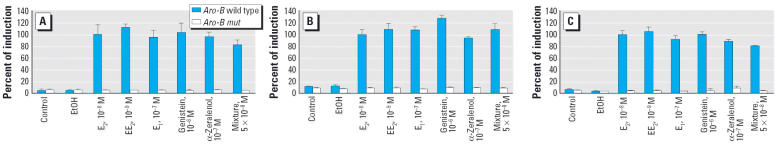
Activation of the *Aro-B* reporter gene by xenoestrogens in U251-MG cells transfected with *Aro-B* wild-type or *Aro-B mut* reporter genes and expression vectors. (*A*) zfER-α. (*B*) zfER-β1. (*C*) zfER-β2. Cells were treated with 0.1% ethanol (EtOH), E_2_, EE_2_, E_1_, genistein, α-zeralenol, or a mixture. Data are expressed as the percentage of induction relative to E_2_ from at least three independent experiments; control represents luciferase activity obtained with empty expression vector.

Even at the highest concentration of all chemicals, we found no luciferase activity without cotransfected ER-α or ER-β expression plasmids, confirming that the transcriptional activity was mediated by ER protein (data not shown). Similarly, we found no luciferase activity with any of the chemicals using the mutated *Aro-B* reporter gene (Figure 5). This clearly indicated that stimulation of luciferase by these chemicals requires direct interaction between ER and the *Aro-B* reporter gene.

### Combination effect of xenoestrogens in the reconstituted glial cell model.

To investigate the mixture effect of E_2_, EE_2_, E_1_, α-zeralenol, and genistein, we determined the ratio for each chemical that should be present in the mixture at an equal potency on the basis of the individual dose–response curves (Kortenkamp and Altenburger 1999; Silva et al. 2002), here at concentrations producing an effect of 30% for ER-α expression. The advantage of this equi-effective design is that all components contributed nearly equally to the overall mixture effect, at least for the ER-α expression and, of course, when the concept of concentration addition holds true. On the other hand, relevant nonchemical interactions may have the chance to become visible and are not masked by the presence of a dominant compound.

**Figure 6:**
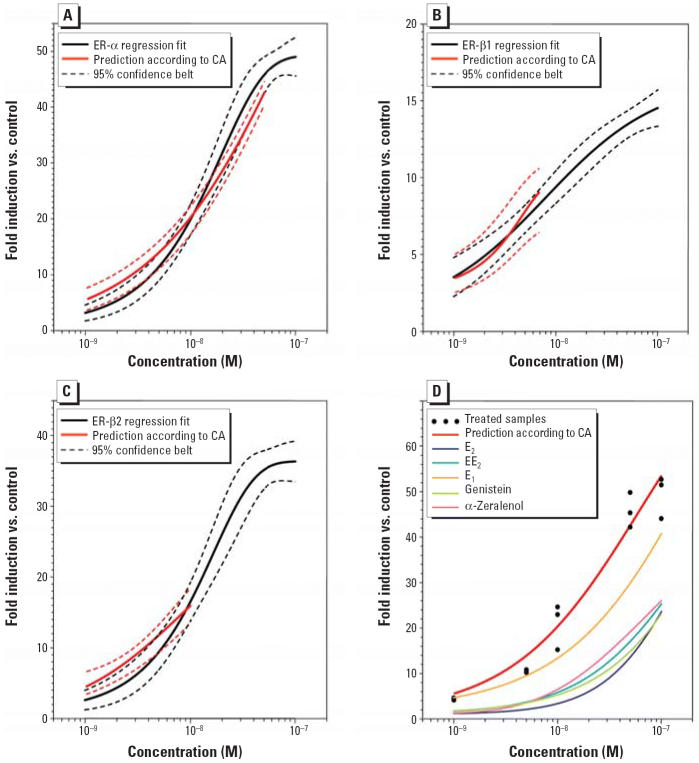
The effect of mixtures of xenoestrogens (E_2_, EE_2_, E_1_, genistein, and α-zeralenol) on the transcriptional activation of zfERs in U251-MG cells transfected with the *Aro-B* reporter gene and expression vectors. (*A*) zfER-α. (*B*) zfER-β1. (*C*) zfER-β2. (*D*) Effects produced with zfER-α by individual components at the concentrations present in the mixture, and the predicted mixture effect calculated according to the concept of concentration addition and the observed mixture effect (treated samples).

We tested the relative potency of this mixture at different concentrations ranging from 1 to 100 nM. A significant high stimulation of luciferase activity was found when the glial cells were treated with increasing concentration of a mixture of the five chemicals, whereas each of those chemicals, at the concentration present in the mixture, is expected to produce only a weak effect if tested singly. As shown in Figure 6, the combination of the five chemicals tested experimentally with ER-α, ER-β1, and ER-β2 showed an additive effect as predicted by the concept of concentration (Rajapakse et al. 2002). However, for ER-β1 and ER-β2 the effect ranges for the predictions are limited: mixture effects can be determined by the concentration addition model only when it is possible to determine for each mixture compound a reliable estimate of a concentration that would produce the same effect when applied on its own. Figure 4 shows that the curve estimates for maximal effects of all tested chemicals differ, for example, with α-zeralenol producing the lowest maximal effect (10%) relative to the controls for ER-β1. Thus, concentrations of α-zeralenol yielding effects > 10% cannot be estimated for this end point, and mixture concentrations corresponding to effects > 10% were impossible to calculate. Thus, Figure 6 demonstrates clearly that the mixture may induce a response that is higher than is possible to induce by one of the compounds. The mixture induced a maximum response of the reporter gene that was about 50-fold with ER-α, whereas each of the chemicals, at a concentration present in the mixture, induced the reporter gene only 8- to 15-fold (Figure 6D).

## Discussion

A current issue for regulatory agencies is to evaluate the potential endocrine-disrupting effects of thousands of chemicals. In particular, estrogenic potency of many environmental persistent chemicals is an important concern for these agencies. At the international level, the consensus recommendation for the assessment of such chemicals is a tiered series of *in vivo* and *in vitro* protocols. With *in vivo* assays, such as rodent uterotrophic assays, vitellogenin assays, or somatic gene transfer into the brain of adult fish (Trudeau et al. 2005), chemicals may be metabolized and may act differently compared with their parental chemicals. However, *in vivo* assays are not suited for the large-scale screening of chemicals because of their cost and complexity and also because these bioassays require the sacrifice of many animals. Moreover, these bioassays are limited for analyzing the molecular mechanisms of action of environmental chemicals. For example, a compound that is a selective ER-β agonist/antagonist would not be expected to show positive effect in tissues that do not express this ER subtype. On the other hand, *in vitro* assays such as ours would be able to identify this compound. Thus, cell-based reporter gene assays are useful means for evaluating the impact of environmental contaminants on the cellular signaling pathways and cellular responses. We and others have developed several *in vitro* bioassays based on mammary, endometrial, hepatic, and yeast models for the characterization of environmental estrogenic chemicals (Ackermann et al. 2002; Andersen et al. 1999; Balaguer et al. 1999; Legler et al. 1999; Le Guevel and Pakdel 2001; Petit et al. 1997; Routledge and Sumpter 1997; Soto et al. 1995).

In this article, we report the development and validation of a new glial cell-based assay providing a fast, reliable, sensitive, and highly responsive test for evaluating the estrogenic or antiestrogenic potency of EDs. We first confirmed that Aro-B is a suitable biomarker to detect the estrogenic potency of chemicals. Indeed, E_2_, EE_2_, E_1_, and α-zeralenol strongly stimulated *Aro-B* gene expression *in vivo*. However, genistein, a well-known phytoestrogen, showed very poor activity. Different reasons could explain this observation, such as stability, transport, and bioavailability. Another explanation, highlighted by our *in vitro* experiments, could be that genistein is more potent for ER-β transcriptional activity. In that case, induction of *Aro-B* might be weak if only ER-α is present in the radial glial cells at this time of zebrafish development or if the ER-α:ER-β ratio is unfavorable. Together, these results show the limitation of such *in vivo* tests that might be overcome by using additional *in vitro* approaches.

One of the advantages of this new cell-based system is that it uses an ER-negative glial cell line. Thus, estrogenic potency of the chemicals can be analyzed on the transcriptional activity of distinct ER subtypes or of a combination of ERs if necessary. Another advantage of this test is that it is based on the use of an endogenous promoter that responds with high efficiency to natural and synthetic estrogens in a glial cell context. A limitation of this assay is that, given the lack of fish glial cell lines, it is based on a heterologous cell context. Nevertheless, we believe that it reflects the *in vivo* situation in fish because *Aro-B* is up-regulated by E_2_ only in radial glial cells *in vivo*. Interestingly, the endogenous Aro-B reporter construct was 3-fold more efficient than the classical ERE-tk-Luc reporter construct commonly used for the screening of estrogenic chemicals. These results suggest that ER may recruit glial-specific factor(s) to mediate E_2_ stimulation of the Aro-B reporter construct. However, all the three ERs did not show similar activity on this reporter gene. In fact, using five potent and structurally different estrogens or xenoestrogens, we found that the highest luciferase activity was achieved with ER-α. The luciferase activity was about 2--fold lower with ER-β2, whereas the luciferase activity was 4- to 6-fold lower with ER-β1.

The mammalian ER-β showed also lower transcriptional activity compared with ER-α in transient transfection experiments using different cell lines and reporter gene constructs (Loven et al. 2001). The reason for that is currently unknown, but it might be due to a differential degradation rate of receptor proteins or a differential stability of the receptor–DNA or receptor–ligand complexes. It might also reflect a differential expression of ER-specific cofactors. Nevertheless, it is interesting to note that, without any ligand, zfER-α consistently stimulated the luciferase activity by 2-fold. This relatively low but significant ligand-independent activity was not observed for ER-β1 and ER-β2. Thus, this glial cell system with ER-β2 showed a detection limit two to five times lower than that for glial cells containing ER-α. At present, it is not clear why ER-α showed a ligand-independent activity in this glial cell context. One reason may be the structural differences in the N-terminal A/B region of zfER-α compared with that of zfER-β subtypes (Menuet et al. 2002). Indeed, this region that was very well characterized as responsible for the ligand-independent activity [ER-α transactivation function 1 (AF-1)] of ERs (Metivier et al. 2000, 2001) and can be regulated by cell-specific factors. The activity of ER AF-1 varies depending upon the target gene and cell type (Merot et al. 2004; Tora et al. 1989; Tzukerman et al. 1994). Additionally, in some cases the activity of AF-1 can be stimulated by phosphorylation in response to growth factors (Kato et al. 1995). The phosphorylation residues may therefore differ among ER-α, ER-β1, and ER-β2. Alternatively, ER-α may be more sensitive than ER-β subtypes to alkylphenols that could be released from plasticware (Soto et al. 1991).

Although the maximum responses with ER-β were weaker than those with ER-α, the EC_50_ values indicate that ER-βs can be more sensitive to some xenoestrogens compared with ER-α. For instance, ER-β2 was 5-fold more sensitive to EE_2_ and 7-fold more sensitive to genistein, compared with ER-α. Interestingly, the phytoestrogen genistein also showed higher binding affinity to the human ER-β, and hence genistein was designed as a SERM (Kuiper et al. 1998). Although this was not our primary objective, the glial cell model described here can also be used for studies examining the activity of SERMs. Of particular interest is the fact that human ER-α can also be used in this system (data not shown). A study with human ER-α and ER-β showed that genistein, for example, has an ER-β-selective affinity and potency but an ER-α-selective efficacy (Barkhem et al. 1998; Kuiper et al. 1998). In addition, tamoxifen and raloxifene have an ER-α-selective partial agonist/antagonist function but a pure antagonist effect through ER-β (Barkhem et al. 1998; Kuiper et al. 1998). Moreover, the agonistic or antagonistic effect of these agents depends on tissue and target–gene contexts (Gustafsson 1998). ER-α and ER-β are able to recruit co-activators (TIF2 and SRC-1a) in the presence of estrogens and some xenoestrogens *in vitro* (Routledge et al. 2000). However, although ER-α and ER-β showed relatively similar binding affinities for the coactivators, the two receptors differed in their ability to recruit the coactivators after xenoestrogen binding.

The presence of low concentrations of estrogenic chemicals in the environment led to the question of whether exposure to weak environmental estrogens can effectively produce adverse hormonal effects in animals and humans (Feldman 1997; Juberg 2000; Safe 1995). In fact, some pesticides as well as alkylphenols, polychlorinated biphenyls, and plastic components act with 100- to 5,000-fold lower potency than E_2_ (Le Guevel and Pakdel 2001; Petit et al. 1997). However, different parameters should be considered: first, relative affinity and effectiveness of xenoestrogens may differ for ER subtypes; second, xenoestrogens may induce different responses depending on cell and promoter context; and third, weakly estrogenic chemicals may act as mixtures in the environment and diet. Using a recombinant yeast model and breast cell lines, Kortenkamp and colleagues (Payne et al. 2000; Rajapakse et al. 2002, 2004; Silva et al. 2002) showed that combining xenoestrogens at levels below individual statistically nonsignificant concentrations may enhance estrogenic effects. These researchers demonstrated that the model of concentration addition is a suitable tool for predicting the mixture effect from the individual activity of each chemical. This model was also confirmed by an *in vivo* study with rainbow trout exposed to binary mixtures of xenoestrogens (Thorpe et al. 2003). In that study, the authors showed that a binary mixture of E_2_ and EE_2_ is more potent than either of the individual chemicals. These data therefore indicate that, for the risk assessment, we should consider the effect of the total estrogenic load of environmental estrogens rather than the individual effect of each chemical. In the present study, we also show that the mixture of five estrogenic chemicals acts in an additive manner in a glial cell model and that the additive action occurs with all three ERs.

In conclusion, because of the complexity of estrogenic signaling pathways, xenoestrogens can act with different mechanisms of action at different levels of organisms. To understand and to evaluate their impact in molecular and cellular aspects of endocrine disruption, it is necessary to develop cell-based transcription assay systems that could reflect different cellular contexts. The assay described here, in addition to being a powerful screening tool, underscores the high sensitivity of the *Aro-B* gene to EDs in a glial cell context. Considering the role of aromatase in brain and sex differentiation of nonmammalian species (Fenske and Segner 2004, Pellegrini et al. 2005), adverse effects could be expected when fish are exposed to EDs during development. Moreover, there is increasing evidence that glial cells are targets of estrogens. However, very little effort has been made to investigate the impact of environmental estrogenic chemicals in glial cells. Here we describe a glial cell model that enables analysis of the impact of environmental estrogenic chemicals on transcriptional activity of all three ER subtypes characterized to date in a vertebrate species. The amount of persistent chemicals has increased over the last 20 years, which highlights the need for high-throughput screening methods. In this glial cell model, the strong E_2_ stimulation of luciferase activity under the control of the E_2_-sensitive Aro-B reporter construct enables accurate results in 96-well plates, making the assay suitable for sensitive and reliable high-throughput screening.

## Figures and Tables

**Table 1:** Potency of different compounds tested in the glial cell system.

Compound, ratio in mix^a^	Receptor	EC_50_ (M)^b^	RSA (%)^c^	RS/ER-α^d^	Maximum induction^e^	LOEC (pM)^f^
E_2_, 0.007	ER-α	1.4 × 10^−9^	27	1.0	70	50–100
	ER-β1	1.9 × 10^−10^	19	7.4	12	10–50
	ER-β2	7.4 × 10^−10^	10	1.9	34	10–50
EE_2_, 0.003	ER-α	3.8 × 10^−10^	100	1.0	56	10–50
	ER-β1	3.7 × 10^−11^	100	10.3	22	1–10
	ER-β2	7.2 × 10^−11^	100	5.3	35	1–10
E_1_, 0.035	ER-α	4.1 × 10^−9^	9	1.0	86	100–500
	ER-β1	7.2 × 10^−9^	0.5	0.6	18	100–500
	ER-β2	2.9 × 10^−8^	0.3	0.1	46	100–500
Genistein, 0.950	ER-α	2.0 × 10^−7^	0.2	1.0	71	5,000–10,000
	ER-β1	5.3 × 10^−7^	0.01	0.4	25	5,000–10,000
	ER-β2	2.9 × 10^−8^	0.2	6.9	25	500–1,000
α-Zeralenol, 0.005	ER-α	5.9 × 10^−10^	64	1.0	79	10–50
	ER-β1	1.1 × 10^−10^	34	5.4	17	10–50
	ER-β2	1.5 × 10^−10^	48	3.9	23	100–500

Abbreviations: LOEC, least observable effect concentration; RS, relative sensitivity; RSA, relative stimulatory activity. All values were determined from data shown in Figures 4 and 6.

a Proportion of each compound in the mixture experiment presented in Figure 6.

b Based on luciferase activity.

c Determined as percentage of estrogenic effect relative to EE_2_.

d Comparison of ER-α, ER-β1, and ER-β2 for different compounds; in all cases, the response with ER-α was arbitrarily set at 1.

e Maximum fold induction of the reporter gene relative to the reporter gene without ERs and compounds.

f The lowest concentration for which 2-fold induction of the reporter gene was obtained.
